# Humoral Immune Response following SARS-CoV-2 Vaccination in Liver Transplant Recipients

**DOI:** 10.3390/vaccines9121422

**Published:** 2021-12-01

**Authors:** Lea Timmermann, Brigitta Globke, Georg Lurje, Moritz Schmelzle, Wenzel Schöning, Robert Öllinger, Johann Pratschke, Bettina Eberspächer, Christian Drosten, Jörg Hofmann, Dennis Eurich

**Affiliations:** 1 Department of Surgery, Charité—Universitätsmedizin Berlin, Corporate Member of Freie Universität Berlin, Humboldt-Universität zu Berlin, and Berlin Institute of Health, 13353 Berlin, Germany; brigitta.globke@charite.de (B.G.); georg.lurje@charite.de (G.L.); moritz.schmelzle@charite.de (M.S.); wenzel.schoening@charite.de (W.S.); robert.oellinger@charite.de (R.Ö.); johann.pratschke@charite.de (J.P.); dennis.eurich@charite.de (D.E.); 2 Labor Berlin—Charité Vivantes GmbH, 13353 Berlin, Germany; bettina.eberspaecher@laborberlin.com (B.E.); joerg.hofmann@charite.de (J.H.); 3 Department of Virology—Universitätsmedizin Berlin, Corporate Member of Freie Universität Berlin, Humboldt-Universität zu Berlin, and Berlin Institute of Health, 13353 Berlin, Germany; christian.drosten@charite.de

**Keywords:** liver transplantation, anti-SARS-CoV-2-antibodies, COVID-19

## Abstract

As COVID-19 remains an issue in transplantation medicine, a successful vaccination can prevent infections and life-threatening courses. The probability of poor immune response in liver transplant recipients gained attention and insecurity among those patients, leading us to investigate the humoral immune response alongside the influence of underlying diseases and immunosuppressive regimen on seroconversion rates. We included 118 patients undergoing anti-spike-protein-IgG testing at least 21 days after completed SARS-CoV-2 vaccination. Ninety-seven patients also underwent anti-spike-protein-IgA testing. The influence of baseline demographics, immunosuppressive regimen and underlying disease on seroconversion was analyzed, and 92 of 118 patients (78.0%) developed anti-spike-protein-IgG antibodies. Patients with a history of alcoholic liver disease before transplantation showed significantly lower seroconversion rates (*p* = 0.006). Immunosuppression also significantly influenced antibody development (*p* < 0.001). Patients run on a mycophenolate mofetil (MMF)-based regimen were more likely not to develop antibodies compared to patients run on a non-MMF regimen (*p* < 0.001). All patients weaned off immunosuppression were seropositive. The seroconversion rate of 78.0% in our cohort of liver transplant recipients is promising. The identification of alcohol-induced cirrhosis as underlying disease and MMF for immunosuppression as risk factors for seronegativity may serve to identify vaccination non-responder after liver transplantation.

## 1. Introduction

The severe acute respiratory syndrome coronavirus 2 (SARS-CoV-2) pandemic not only influences social life and daily health care work, but especially transplantation medicine all over the world. The mortality of all hospitalized solid organ transplant recipients suffering from coronavirus disease 19 (COVID-19) is reported to be 20% [1]. This is consistent with reported mortality rates of 12–20% for liver transplant (LT) recipients in general and rates of 17–28% in cases of hospitalization. [2,3,4]. Still, a recent multicenter study showed that LT as a risk factor did not significantly increase mortality in patients with COVID-19 [5]. Nevertheless, even for otherwise healthy individuals without immunosuppression, COVID-19 may constitute a life-threatening issue and consecutively influence the quality of life. With no proper treatment option at hand, vaccination soon became the ray of hope not only in the general population. Early and sufficient vaccination might also prevent life-threatening infections in solid organ transplant recipients under immunosuppressive therapy. However, there is little evidence regarding the immune response following a completed SARS-CoV-2 vaccination in solid organ transplant recipients and even less evidence is presented for LT recipients in particular. First results reporting a poor humoral response rose insecurity among those patients and their practitioners and led to questions of early booster vaccinations and change of vaccination substrate. It remains unclear how the humoral immune response as well as specific T-cell immunogenicity affect patients’ actual protection from infection in the first place or from a severe course of disease, respectively. Villanego et al. reported on a rate of 1.8% of completely vaccinated kidney transplant recipients developing a breakthrough infection and consecutively COVID-19 [6], whereas Aslam et al. reported on an even lower rate of 4 out of 912 solid organ transplant recipients [7]. Malinis et al. reported 0.65% of their fully vaccinated solid organ transplant recipients developed COVID-19 [8]. Immunosuppressive therapy, however, may not only advance the risk of SARS-CoV-2 infections and the severity of the course of disease. It might also influence seroconversion and protection following completed vaccination. Transplant center outreach to encourage at least basic vaccination is an important point to take into consideration, especially in the early phase [9]. Thus, the objective of the study was to determine the seroconversion rate after complete vaccination and to identify risk factors for non-response.

## 2. Materials and Methods

### 2.1. Patients, Inclusion and Exclusion Criteria

**Figure 1:**
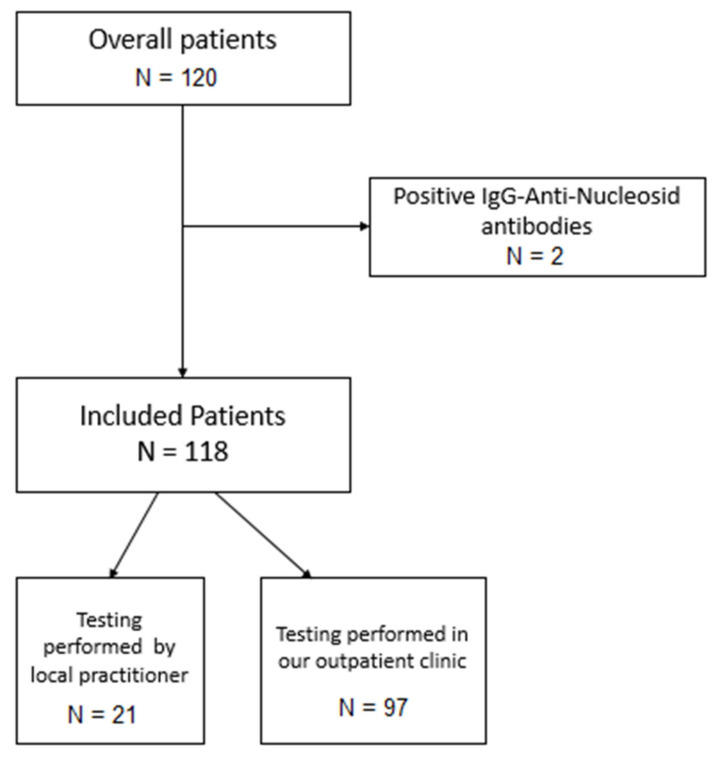
Inclusion process. After exclusion of 2 asymptomatic patients with positive IgG-anti-nucleocapsid antibodies an overall of 118 patients, 97 of them tested at our outpatient clinic and 21 tested by a local practitioner, were included for further analysis.

Between May and July 2021, 120 LT recipients underwent SARS-CoV-2 antibody testing at least 21 days after completed SARS-CoV-2 vaccination. Testing was either performed in our outpatient clinic (*n* = 99) or by local practitioners with transfer of the results to our transplant center (*n* = 21). Completed vaccination was defined according to the recommendation of the respective vaccine. Patients undergoing vaccination past clinical apparent or otherwise confirmed infection with SARS-CoV-2 were not included for further analysis. Within the initial study cohort, we identified two asymptomatic patients with anti-nucleocapsid-IgG antibodies and excluded them from further analysis. All included patients are regularly treated at our outpatient clinic in a lifelong after-care program regarding, e.g., liver transplant function, development of comorbidities and adjustment of the immunosuppressive therapy. Each immunosuppressive regimen is thereby individually updated considering the underlying disease, history of rejection and current drug level as well as apparent or expected side effects. Each individual included in this study made personal contact regarding their vaccination with one of the investigators. Additional information regarding patients’ characteristics and level of immunosuppression was retrieved from our clinic’s database and electronic health care records by one of the investigators. One hundred and fourteen of all patients had undergone completed vaccination with the mRNA-based vaccine BNT162b2 (BioN-Tech Manufacturing GmbH/Pfizer, Mainz, Germany), three with the mRNA-based vaccine mRNA-1273 (Moderna, Cambridge, MA, USA) and one with the vector-based vaccine JNJ-78436735 (Janssen Pharmaceuticals, Companies of Johnson & Johnson, New Brunswick, NJ, USA). Patients tested at our outpatient clinic (*n* = 97) also underwent qualitative and quantitative testing of anti-spike-protein-IgA antibodies. Figure 1 indicates the inclusion process. As soon as our country’s transplant society published their vaccination recommendations for solid organ transplant recipients, we sent recommendation letters to our LT recipients in order to certify their prioritization in the general vaccination order and to encourage our patients and their local practitioners to perform full vaccination at an early stage. Vaccination was performed either by local practitioners or a local vaccination center. Vaccination was also recommended to our patients’ relatives to prevent break-through infections at an early stage.

### 2.2. Antibody Assessment

In our center, serum samples were tested using the Elecsys Anti-SARS-CoV-2 assay (Roche Diagnostics, Mannheim, Germany) for nucleocapsid total antibodies and Anti-SARS-CoV-2-Elisa (Euroimmun, Lübeck, Germany) for IgG and IgA to the viral spike protein according to the manufacturer’s instructions and presented as ratios. The ratio is defined as a dimensionless unit in which the quantitative level of IgG and IgA antibody response are measured. It is calculated as the quotient of the extinction value of the patient sample and the calibrator. For both IgG and IgA, local practitioners used approved commercially available ELISA assays. Results from these 21 cases were excluded from quantitative analysis and were only included in the initial qualitative analysis of seroconversion due to lacking comparability of absolute levels at the time the study was performed.

### 2.3. Statistical Analysis

Data were processed using SPSS version 27.0 (IBM, Armonk, NY, USA). Two-tailed Pearson’s chi-square test was performed on categorical and ordinal scaled data, and a Mann–Whitney-U-test and Kruskal–Wallis-test were performed on interval scaled data. Significance tests were two-sided, and *p* < 0.05 was considered to be statistically significant.

## 3. Results

### 3.1. Results from the Overall Cohort

In the early phase of the vaccination process we actively encouraged our patients to get vaccinated. At the same time, we subjectively observed a high intrinsic motivation in our LT recipients. We included an overall of 118 patients for further analysis. One hundred and fourteen of them had undergone completed vaccination with the mRNA-based vaccine BNT162b2 (BioN-Tech Manufacturing GmbH/Pfizer, Mainz, Germany), three with the mRNA-based vaccine mRNA-1273 (Moderna Biotech Spain, Madrid, Spain) and one with the vector-based vaccine JNJ-78436735 (Janssen Pharmaceuticals, Companies of Johnson & Johnson, New Brunswick, NJ, USA). The mean interval between vaccination completion and blood sampling was 44.6 days (21–132 days). Ninety-two of one hundred and eighteen patients (78.0%) had developed detectable SARS-CoV-2-specific anti-spike-protein-IgG antibodies. As indicated above, all patients were seronegative for anti-SARS-CoV-2-nucleocapsid-IgG antibodies. Table 1 summarizes results from the overall cohort. Twenty patients (76.9%) without and fifty-five patients (59.8%) with seroconversion were male and six patients (23.1%) without and thirty-seven patients (40.2%) with seroconversion were female (*p* = 0.109). Neither time between transplantation and vaccination, with a mean of 14.8 years (0–32) in the IgG positive group and a mean of 12.9 years (0–3; *p* = 0.186) in the IgG negative group nor the age at vaccination, with a mean of 65.1 years (28–84) in the IgG positive and of 69.4 years (42–89; *p* = 0.232) in the IgG negative group were significantly different. The distribution of the primary disease leading to transplantation differed significantly between seropositive and -negative patients (*p* = 0.006). Additionally, 46.2% of all seronegative patients had an alcohol-induced liver disease (ALD) as underlying disease, whereas for the seropositive cohort only 14.1% were transplanted because of ALD. Further, 7.7% of seronegative and 28.3% of seropositive patients received a transplant because of viral end stage liver disease, and 26.9% of the patients in the IgG negative group compared to 20.7% in the IgG positive group underwent transplantation because of malignant disease. We found that 11.5% of the IgG negative individuals had an autoimmune disorder as their primary disease; in the IgG positive group the rate was 16.3%. The influence of the applied immunosuppressive regimen was also significant (*p* < 0.001). All patients currently not treated with any immunosuppressive medication developed anti-spike-protein-IgG-antibodies; 73.1% of all seronegative patients were run on a mycophenolate mofetil (MMF)-based regimen, whereas 22.8% of the seropositive patients were treated with MMF. This finding appears to be statistically significant (*p* < 0.001). Treatment with calcineurin inhibitors did not show a significant effect on seroconversion rates.

### 3.2. Anti-Spike-Protein-IgG- and IgA-Levels Corresponding Immunosupression and Underlying Disease

**Figure 2:**
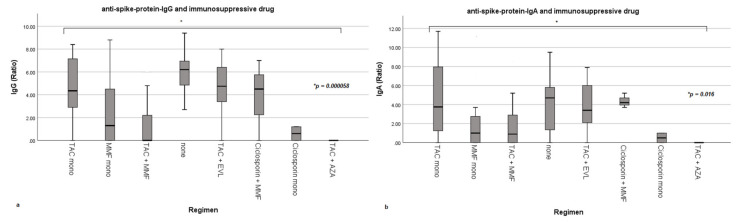
Influence of immunosuppressive drugs on anti-spike-protein-IgG- and IgA-levels as box plots. (**a**,**b**) show the influence of the immunosuppressive regimen on anti-spike-protein-IgG and IgA-levels. MMF = mycophenolate mofetil; TAC = tacrolimus, EVL = everolimus; AZA = azathioprine. Ratio = dimensionless unit; quotient of the extinction value of the patient sample and the calibrator. Box plots present means, interquartile range (IQR), minimum and maximum with each individual bar.

As quantitative levels for both anti-spike-protein-IgG and IgA were only available for patients tested at our outpatient clinic, the following analysis was conducted on this cohort (*n* = 97). Table A1 (Appendix A) indicates comparability of results regarding baseline characteristics from patients from our outpatient clinic to the overall cohort. As Figure 2 indicates, the immunosuppressive regimen significantly influences both anti-spike-protein-IgG (*p* = 0.000058) and IgA levels (*p* = 0.016). The highest levels for both IgG and IgA were reached in the group weaned off any immunosuppression followed by the group receiving a tacrolimus monotherapy.

**Figure 3:**
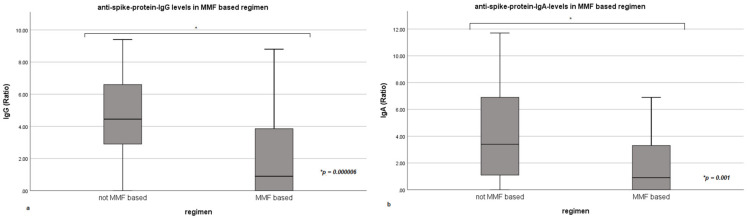
Influence of mycophenolate mofetil (MMF)-based immunosuppression on anti-spike-protein-IgG- and IgA-levels as box plots. (**a**,**b**) show the influence of mycophenolate mofetil based immunosuppression on anti-spike-protein-IgG- and IgA-levels. Ratio = dimensionless unit; quotient of the extinction value of the patient sample and the calibrator. Box plots present means, interquartile range (IQR), minimum and maximum with each individual bar. Box plots present means, interquartile range (IQR), minimum and maximum with each individual bar.

Anti-spike-protein-IgG (*p* = 0.000006) and IgA-levels (*p* = 0.001) were significantly lower in patients run on a MMF-based regimen (compare Figure 3). This includes both mono- and combination therapy.

**Figure 4:**
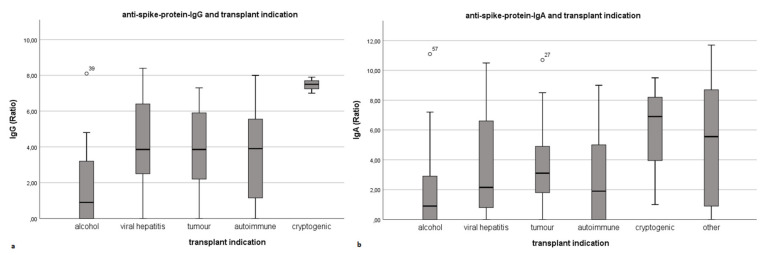
Influence of transplant indication on anti-spike-protein-IgG- and IgA-levels as box plots. (**a**,**b**) show the significant influence of the underlying disease on anti-spike-protein-IgG- and IgA-levels. Ratio = dimensionless unit; quotient of the extinction value of the patient sample and the calibrator. Box plots present means, interquartile range (IQR), minimum and maximum with each individual bar.

As Figure 4 indicates, the underlying disease significantly influences the level of anti-spike-protein-IgG (*p* = 0.005). Antibody levels in patients with alcohol-induced liver cirrhosis prior to LT were significantly lower compared to other LT indications. In comparison, anti-spike-protein-IgA levels also differed regarding the underlying disease and showed a similar pattern, although the findings for anti-spike-protein-IgA did not reach statistical significance (*p* = 0.051).

Thirteen of the twenty-two IgG-negative patients (59.1%) also tested negative for anti-spike-protein-IgA. Three of them were not run on MMF-based immunosuppression. Nine IgG-negative patients were tested IgA-positive, of which three patients were also not run on MMF-based immunosuppression. IgG- and IgA-levels were significantly correlated (*p* < 0.001). Time from vaccination completion to antibody testing (in days) neither significantly correlated with the overall anti-spike-protein-IgG positivity nor with the anti-spike-protein-IgG- and IgA-levels. To the best of our knowledge, none of the patients having undergone completed SARS-CoV-2 vaccination tested positive or developed clinically apparent COVID-19. No severe adverse reactions to the vaccine were reported.

## 4. Discussion

The humoral immune response by means of anti-spike-protein-IgG positivity in our homogenous cohort of LT- recipients appears high compared to previously published study results. Boyarsky et al. were the first to report on humoral immune response following a single dose of mRNA-based vaccines in a heterogenous group of solid organ transplant recipients. They detected a seroconversion rate of 17% for the overall cohort and of 37% for LT recipients, respectively [10]. In a follow-up study performed on a larger cohort and after completed vaccination, 15% of all participants had developed antibodies both after the first and second mRNA-based vaccination (BNT162b2, mRNA-1273), whereas 39% reached seroconversion after the second vaccination only and 46% remained seronegative after completed vaccination. In this group 48% of the LT recipients reached seroconversion after having received the second mRNA-based vaccination, 32% had antibodies both after the first and second vaccination and 20% remained seronegative after the second vaccination [11]. These findings are consistent with our results of a seroconversion rate of almost 80%. Marion et al. reported on data of 367 patients, 58 of them following LT, four weeks after completed vaccination. In 34% of all solid organ transplant recipients and 50% of LT recipients anti-spike-protein-IgG antibodies were detected [12]. Rabinowich et al. showed a seroconversion rate of 47.5% following mRNA-based vaccination and identified MMF based immunosuppression as a risk factor for seronegativity [13]. The variance in seroconversion rates published so far is most certainly caused by each center’s immunosuppression regimen and aimed base levels. Supporting these findings, kidney transplant recipients, whose immunosuppression tends to be stricter and on a higher level, reached even lower seroconversion rates of 22–49% [14,15,16]. In our cohort, seronegative patients tended to be older and more often male, although these findings did not reach statistical significance. In their multiple-cohort analysis, Glatman-Freedman et al. report the vaccine effectiveness to develop slower in individuals of 80 years and older [17]. The underlying disease seemed to have a significant influence on seroconversion rates in our cohort. Patients with alcohol-induced cirrhosis before transplantation were more likely not to develop antibodies and reached significantly lower quantitative levels of anti-spike-protein-IgG and IgA. Long-lasting effects of ethanol-metabolite toxicity not only to the liver, but also e.g., the bone marrow and consecutively the immune system may serve as an explanation. However, these findings may have been influenced by the applied immunosuppressive regimen, although the applied regimen for ALD patients is significantly less strict compared to LT recipients with an autoimmune disease as LT-indication. Nevertheless, further studies are mandatory to confirm that a history of a chronic alcohol abuse and its aftereffects are an independent risk factor, not only in the context of a performed transplantation. In a prospective study Thuluvath et al. compared the immune response of LT recipients to those suffering from cirrhosis and chronic liver diseases without cirrhosis four weeks after the second dose of SARS-CoV-2 vaccination. They found a rate of undetectable antibodies in 17.8% of the LT recipients, 3.8% of the patients with cirrhosis and 4.3% of the patients with chronic liver disease without cirrhosis. An overall of 61.3% of the LT recipients and 24% of the patients with chronic liver diseases in this study developed either a poor antibody response or none at all [18]. In our cohort, no serious adverse reactions to the vaccine occurred. Dumortier reports on a single case of transient liver injury occurring in temporal context with a first mRNA-based vaccination [19]. The applied immunosuppressive regimen also significantly influenced the seroconversion rate and level of both anti-spike-protein-IgG and IgA levels in our study. All patients completely weaned off immunosuppressive medication were seropositive, whereas a MMF-based regimen significantly decreased seroconversion rates and lowered antibody levels in our cohort. Obviously, the extent of immunosuppression also matters in the field of a successful SARS-CoV-2 vaccination. MMF was identified to significantly influence seroconversion in this field. In the daily practice of immunosuppression, the use of MMF, especially as a combination therapy, is usually performed for two reasons: firstly to intensify immunosuppression, e.g., for suspected or manifest rejection, and secondly to balance it on T and B cells. The second is frequently the case in liver transplant patients, which explains the difference in kidney transplant patients and supports the previously published results. Immunosuppression containing MMF has also been described to be an independent predictor for severe COVID-19 in LT recipients, whereas calcineurin inhibitors did not show a significant effect [4]. However, all these findings require an adequate response regarding questions of booster vaccinations or change in immunosuppressive therapy. Another factor that should be taken into consideration is the adequate time of vaccination. Grupper et al. compared seroconversion rates of patients undergoing completed vaccination prior to kidney transplantation to those having received their completed vaccination after kidney transplantation. They found that in the group undergoing vaccination prior to their kidney transplantation, a seroconversion rate of 90% was achieved compared to 45% in the group undergoing vaccination past their kidney transplantation [20]. In our cohort all patients received their vaccination past their transplantation. We recommended they receive the first dose at least 6 months after transplantation to increase the chance of seroconversion and protection and minimize the chance of adverse effects following the recommendations for common vaccinations after liver transplantation. Kamar et al. showed an increase in humoral response following a third mRNA-based vaccination in solid organ transplant recipients [21], but large-scale data, not only for LT- recipients, are still missing. Del Bello et al. report on their cohort of solid organ transplant recipients undergoing a third mRNA-based vaccination. They found seropositivity of 41.4% after the second dose and an increase to 67.9% after the third dose. They additionally report that all patients seropositive before the third vaccination remained positive four weeks after the third dose [22]. A recent study confirmed that kidney and liver transplant recipients having undergone SARS-CoV-19 infection and consecutively having developed anti-spike-protein-IgG antibodies remained seropositive in a mean follow-up of 4.9 months [23]. We performed testing at a mean of 44.6 days after completed vaccination, a period after which cited studies suggest maintenance of seropositivity. Serum concentration of IgA antibodies, which are naturally distributed over mucosal surfaces, has been proven to decline earlier compared to IgG serum levels [24,25]. However, the importance of the quantitative level of circulating antibodies, IgG and IgA, to this respiratory infection remains unclear, taking issues such as t-cell-mediated immunity and the definition of neutralizing antibodies into account. Ruether et al., in whose cohort of LT recipients a seroconversion rate of 63% regarding anti-spike-protein-IgG was achieved, detected a spike-specific T-cell response in 36.6% in the same cohort compared to a cohort of patients with liver cirrhosis and healthy controls, who both developed anti-spike-protein-IgG in 100% of the cases and had a spike-specific T-cell response in 65.4 and 100% of the cases, respectively [26]. Westhoff et al. detected a seroconversion rate of 60% and anti-spike-specific T-cells in 90% of kidney transplant recipients following a third dose of mRNA-based vaccine following initial humoral nonresponse following the second dose [27]. Hall et al. detected a low humoral response rate of anti-spike-protein antibodies of 5% following the first dose and of 34.5% following the second dose of mRNA-based vaccination in their heterogeneous group of solid organ transplant recipients. The subgroup of LT recipients developed the highest rate compared to the others. They observed specific CD4+ T-cells to be found in 10% of the individuals following the first dose and in 47.9% following the second dose. Specific CD8+ T-cells were not detected at all. Additionally 47.8% of the patients with detectable CD4+ T-cells also had detectable anti-spike-protein antibodies [28]. Sattler at al. only found 2.6% of their kidney transplant recipients to be seropositive for anti-spike-protein-IgG following a second dose of mRNA-based vaccination after a mean period of 8 days. They furthermore found a seroconversion rate of 10.26% regarding anti-spike-protein-IgA. The rates slightly changed to 8.33% for IgG and 13.04% for IgA after an additional control was made at a later timepoint. However, a high rate of specific CD4+ T-cells was found, whereas a low rate (5.13%) was found for specific CD8+ T-cells [29]. Based on current knowledge measurement of T-cell-mediated protection, this underlies a high variance. As long as the role of both the humoral and the cellular immune response following SARS-CoV-2-vaccination for solid organ transplant recipients remain unclear, maintenance of meticulous hygiene and vaccination of patients’ environment is essential. However, another factor to be considered is that such systemic infections can induce autoimmune diseases. Additionally, patients suffering from autoimmune diseases in some studies were equally or slightly more affected by COVID-19 [30]. In our cohort, seroconversion rates of patients undergoing liver transplantation due to autoimmune disease developed comparable high amounts of specific antibodies especially compared to other subgroups. The significant effect of underlying disease and immunosuppressive regimen was seen for both IgG and IgA. However, further studies are mandatory to evaluate the advantage of a third dose for patients presenting the identified risk factors. Immediate help and the key to success regarding the seroconversion of LT recipients following completed SARS-CoV-2 vaccination in our opinion based on current knowledge is a careful and foresighted handling of immunosuppressive therapy. As not only side effects such as chronic kidney insufficiency and the increased incidence of de novo malignoma can be reduced with a deliberate use of immunosuppression, in our opinion, an individually tailored approach to immunosuppressive therapy may be particularly important in today’s pandemic. The current study is limited by common biases mainly due to its retrospective character.

## 5. Conclusions

At almost 80%, the humoral response to a completed SARS-CoV-2 vaccination in LT- recipients was high in our cohort. We identified alcohol-induced cirrhosis as a risk factor for seronegativity. Patients seem to benefit from a low level of immunosuppressive therapy. As overimmunosuppression, alongside other issues, impairs vaccination results, the extend of immunosuppressive therapy should be carefully re-evaluated in the individual patient. MMF in particular has a negative influence on the humoral immune response following SARS-CoV-2-vaccination in LT- recipients.

## Figures and Tables

**Table 1:** Patients’ characteristics in the overall cohort.

	Overall Cohort (N = 118)	IgG Positive (N = 92)	IgG Negative (N = 26)	*p*-Value
Sex N (%)				0.109
Male	75 (63.6)	55 (59.8)	20 (76.9)	
Female	43 (36.4)	37 (40.2)	6 (23.1)	
Time since transplantation (years)				0.186
Mean	14.4	14.8	12.9	
Minimum	0	0	0	
Maximum	37	32	37	
Age at vaccination (years)				0.232
Mean	66.1	65.1	69.4	
Minimum	28.0	28	42	
Maximum	89	84	89	
Transplant indication N (%)				0.006
Alcohol-induced	25 (21.1)	13 (14.1)	12 (46.2)	
Viral hepatitis	28 (23.7)	26 (28.3)	2 (7.7)	
Tumor	26 (22)	19 (20.7)	7 (26.9)	
Autoimmune	18 (15.3)	15 (16.3)	3 (11.5)	
Cryptogenic	4 (3.4)	4 (4.3)	0 (0)	
Other	17 (14.4)	15 (16.3)	2 (7.7)	
Immunosuppression N (%)				0.000270
Tacrolimus mono	42 (35.6)	40 (43.5)	2 (7.7)	
MMF mono	16 (13.6)	10 (10.5)	6 (23.1)	
Tacrolimus + MMF	24 (20.3)	12 (13)	12 (46.2)	
Tacrolimus and Everolimus	15 (12.7)	12 (13)	3 (11.5)	
Everolimus mono	1 (0.8)	1 (1.1)	0 (0.0)	
Ciclosporin + MMF	3 (2.5)	2 (2.2)	1 (3.8)	
Ciclosporin mono	2 (1.7)	1 (1.1)	1 (3.8)	
None	14 (11.9)	14 (15.2)	0 (0)	
Tacrolimus + Azathioprin	1 (0.8)	0 (0)	1 (3.8)	
MMF-based regimen	40 (33.9)	21 (22.8)	19 (73.1)	*0.000002*

MMF = mycophenolate mofetil.

## Data Availability

Data from this study are available from the corresponding author on reasonable request.

## Appendix A

**Table A1:** Results from outpatient clinic.

	Overall Cohort (N = 97)	IgG Positive (N = 75)	IgG Negative (N = 22)	*p*-Value
Sex N (%)				0.193
Male	59 (60.8)	43 (57.3)	16 (72.2)	
Female	38 (39.2)	32 (42.7)	6 (27.3)	
Time since transplantation (years)				0.244
Mean	13.5	14.0	12.0	
Minimum	0	0	0	
Maximum	32	32	30	
Age at vaccination (years)				0.425
Mean	65.7	65.0	68.1	
Minimum	31	31	42	
Maximum	84	84	82	
Transplant indication N (%)				0.046
Alcohol-induced	21 (21.6)	11 (14.7)	10 (45.5)	
Viral hepatitis	22 (22.7)	20 (26.7)	2 (9.1)	
Tumour	22 (22.7)	17 (22.7)	5 (22.7)	
Autoimmune	16 (15.5)	12 (16.0)	3 (13.6)	
Cryptogenic	3 (3.1)	3 (4.0)	0 (0)	
Other	14 (14.4)	12 (16.0)	2 (9.1)	
Immunosuppression N (%)				0.001
Tacrolimus mono	32 (33.0)	30 (40)	2 (9.1)	
MMF mono	13 (13.4)	9 (12.0)	4 (18.2)	
Tacrolimus + MMF	21 (21.6)	10 (13.3)	11 (50)	
Tacrolimus and Everolimus	14 (14.4)	12 (16.0)	2 (9.1)	
Everolimus mono	0 (0)	0 (0)	0 (0)	
Ciclosporin + MMF	3 (3.1)	2 (2.7)	1 (4.5)	
Ciclosporin mono	2 (2.1)	1 (1.3)	1 (4.5)	
None	11 (11.3)	11 (14.7)	0 (0)	
Tacrolimus + Azathioprine	1 (1)	0 (0)	1 (4.5)	
MMF-based regimen	35 (36.1)	19 (25.3)	16 (72.2)	0.000047

MMF = mycophenolate mofetil.
